# Supplementary material for: Mechanobiologically-optimized non-resorbable artificial bone for patient-matched scaffold-guided bone regeneration
Source: Nat Commun. 2025 Oct 24;16:9422. doi: 10.1038/s41467-025-64466-z (PMC12552697; doi:10.1038/s41467-025-64466-z)
Supplement: Supplementary file 1 — Supplementary Information [file 41467_2025_64466_MOESM1_ESM.pdf]

Supplementary Information

Mechanobiologically-optimized non-resorbable artificial bone – a new paradigm in patient-matched scaffold-guided bone regeneration

Jonathan R. Clark[1,2,3,4,5]*, D S Abdullah Al Maruf[2,3], Eva Tomaskovic-Crook[6,7,8], Kai Cheng[2,4], William T. Lewin[6,8,9], Hai Xin[2], Boyang Wan[1,10], Jiongyu Ren[1,11], Chi Wu[10], Hedi V. Kruse[6,9,12], Daniel K. Lawrence[13], Innes Wise[14], Aditi Gupta[1,2], Maria A. Woodruff[1,15], Maryam Alsadat Rad[6,8], David Leinkram[2,3,5], Timothy Manzie[1,2,3,5], Krishnan Parthasarthi[2,3,5], James Wykes[2,3,5], Tsu-Hui (Hubert) Low[2,3,3,16], Dale Howes [2,17], Catriona Froggatt[2,5], Ruta Gupta[1,3,18], Gordon Wallace[1,7], Qing Li[1,10,19], David R. McKenzie[6,9,12], Jeremy M. Crook[1,6,7,8,9]*

*Corresponding Authors: jonathan.clark@lh.org.au, jeremy.crook@lh.org.au

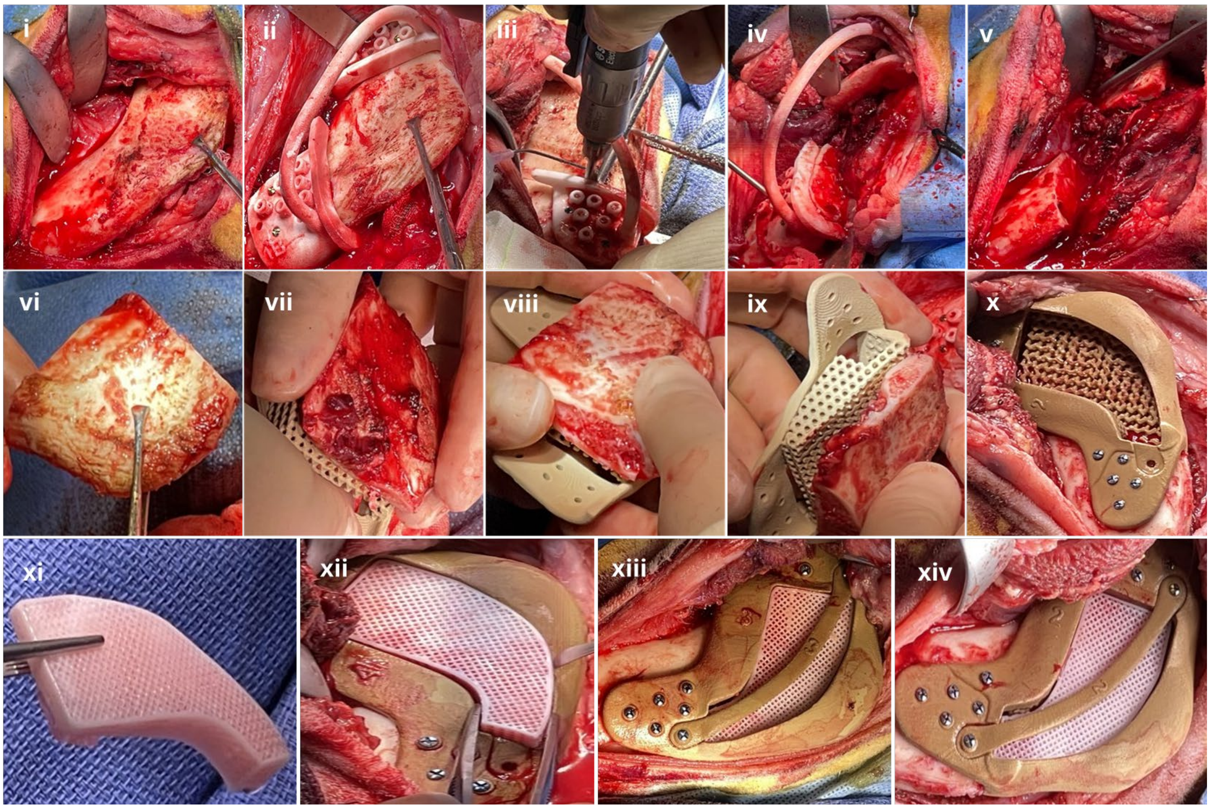

Supplementary Fig. 1: Sequential intraoperative steps showing the process of scaffold implantation in Sheep following a segmental mandibulectomy.

(i–ii) The left mandible was surgically exposed, and the sheep-specific cutting guide was positioned and secured in place. **(iii)** A reciprocating saw was used to perform the osteotomy through the guide, creating a segmental mandibulectomy defect. **(iv–v)** The defect was visualized after completion of the cut and removal of the cutting guide. **(vi)** The excised bone segment was retrieved. **(vii-ix)** The excised segment was compared to the customized LS-PEK (laser sintered polyether ketone) scaffold to confirm anatomical fit. **(x)** LS-PEK frame and scaffold being fixated to the left mandible. **(xi)** A βTCP (beta-tricalcium phosphate) lattice was infused with ADSC-laden (adipose-derived stem cell) GelMA hydrogel for regenerative support. **(xii)** The cell-laden βTCP lattice was carefully inserted into the cavity within the LS-PEK frame. **(xiii-xiv)** LS-PEK frame with βTCP lattice held in place by LS-PEK crossbar before wound closure. Sample size: n=5.

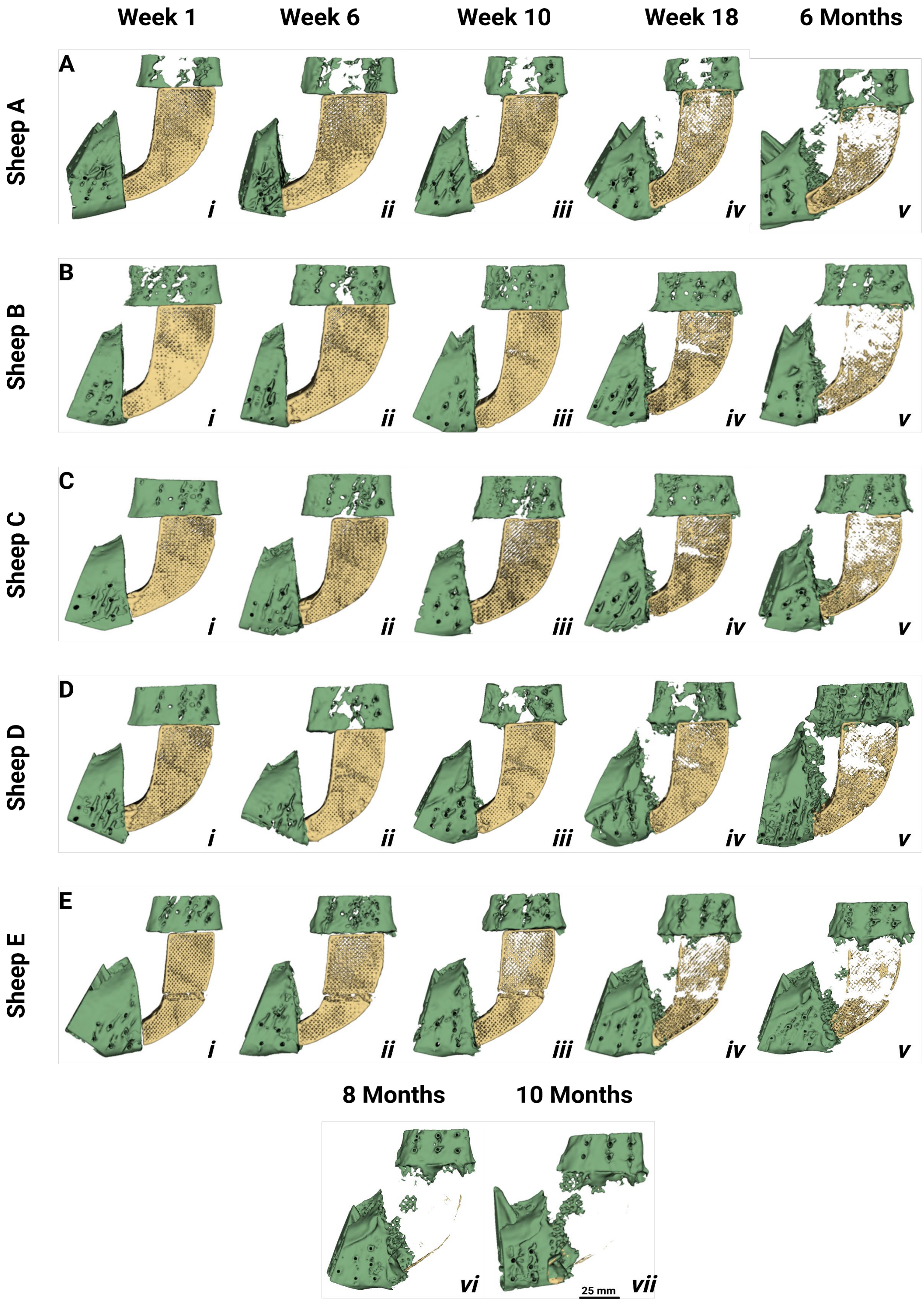

Supplementary Fig. 2: 3D reconstruction of new bone formation within the hybrid artificial bone from CBCT scans taken at various time intervals. This figure shows longitudinal 3D CBCT reconstructions tracking bone formation in segmental mandibular defects of five sheep (Sheep A-E) at various time points post-implantation: Baseline (i), Week 6 (ii), Week 10 (iii), Week 18 (iv), and 6 Months (v). Panels vi and vii display additional scans at 8 and 10 months for Sheep E. Progressive new bone (green) was formed and shown to be interlocking with the LS-PEK frame (which is radiolucent) in all sheep, however notable osteoconduction bridging the defect was only observed in Sheep A, D, and E. The βTCP lattice within the segmental defect is radio-opaque (yellow). Islands of new bone (green) are seen within the βTCP lattice, which progressively degrade over time but at different rates within each sheep. Scale bar: 25 mm. Sample size: n=5.

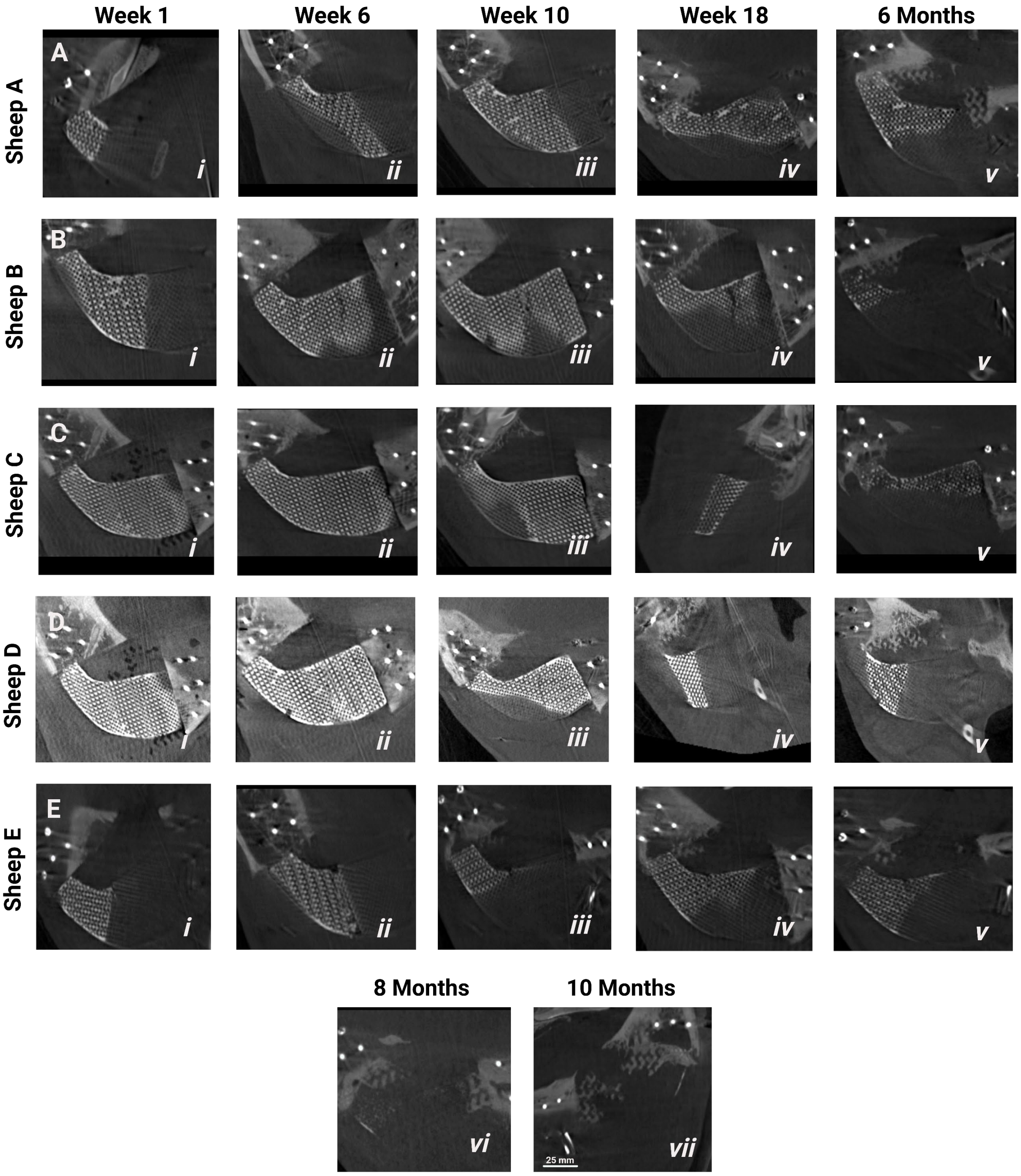

Supplementary Fig. 3: Selected 2D sagittal images of new bone formation within the hybrid artificial bone from CBCT scans taken at various time intervals. CBCT scans display progressive bone formation within the hybrid artificial bone in five individual sheep (A-E) across various post-operative time points: Week 1 (i), Week 6 (ii), Week 10 (iii), Week 18 (iv), and 6 Months (v). Additional time points of 8 Months (vi) and 10 Months (vii) are presented for Sheep E. The 2D images more clearly show new bone filling the voids of the LS-PEK gyroid scaffold structure than the 3D images; this is evident in all sheep at the bone-implant interface, but can be seen more extensively in sheep A, D, and E. Sample size: n=5

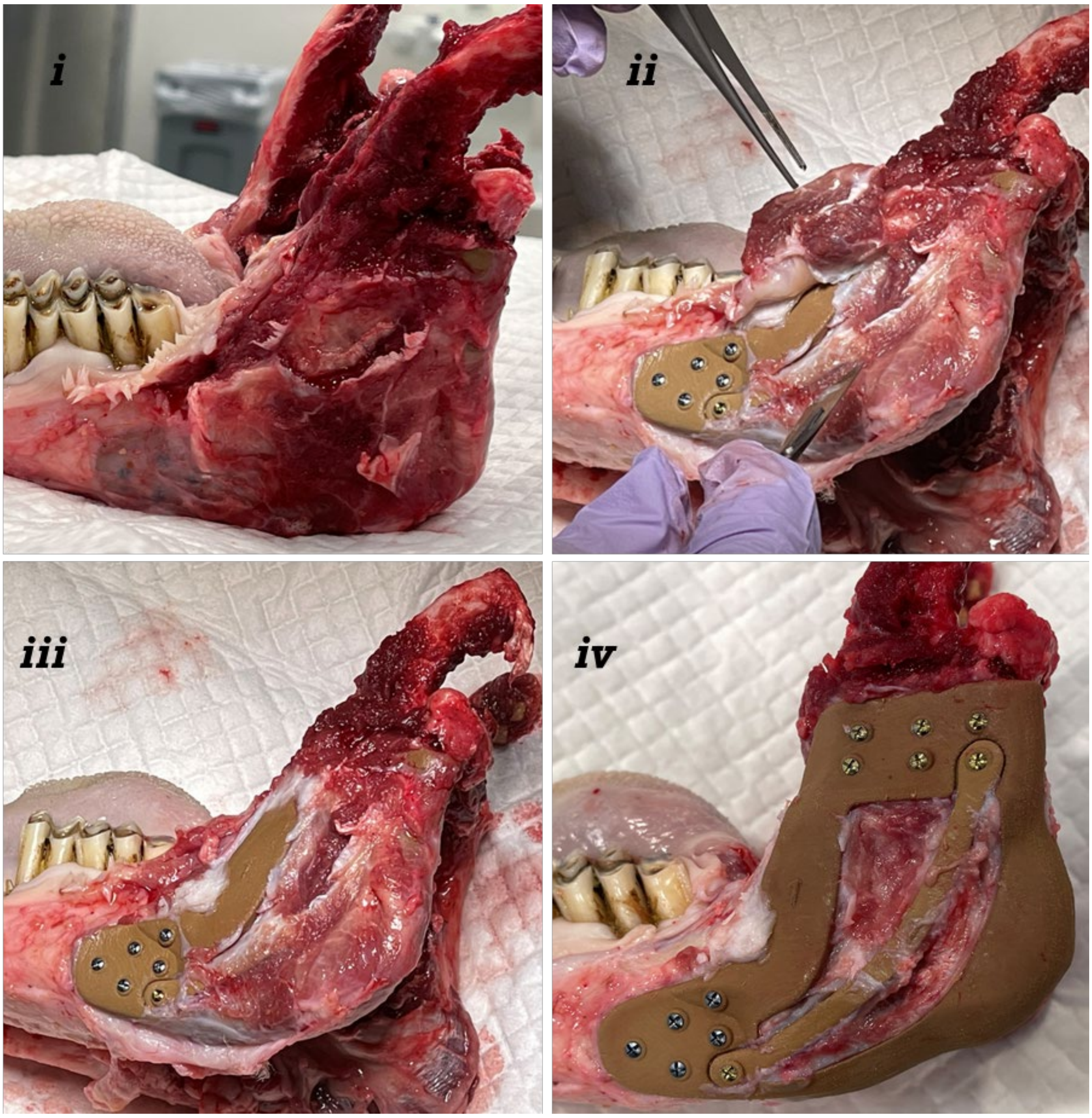

Supplementary Fig. 4: Sequential removal of muscle tissue from the scaffold at the time of sacrifice. **i.** Overview of the explanted mandible and associated muscle tissue immediately post-sacrifice. **ii.** Partial removal of muscle tissue exposes the underlying scaffold. **iii.** Further dissection. **iv.** Complete removal of surrounding muscle tissue, showing the entire scaffold and the underlying bone interface. Sample size: n=5

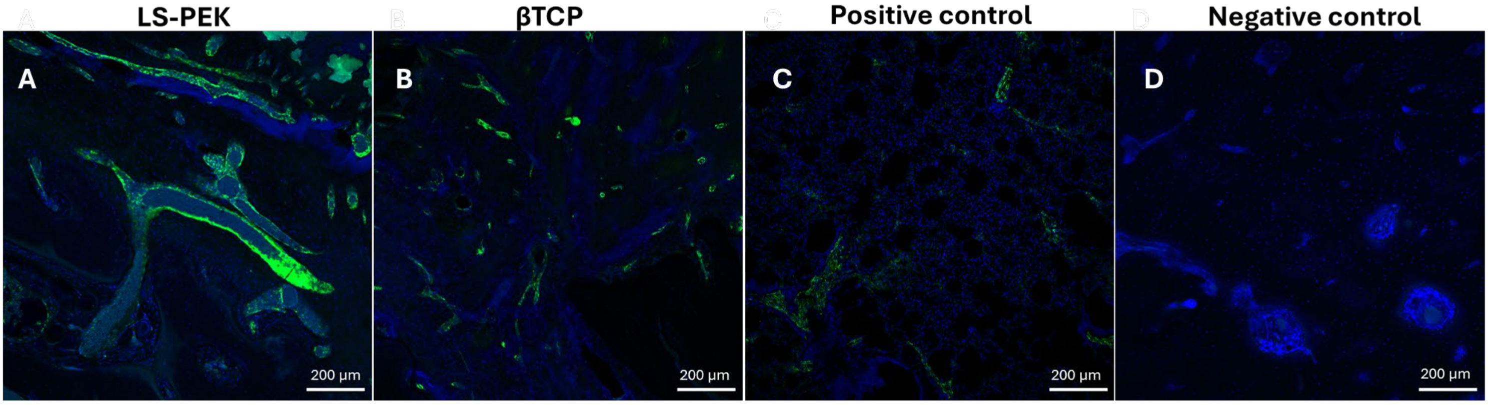

Supplementary Fig. 5: Immunofluorescence for neovascularization: Staining for von Willebrand Factor (vWF), a marker of endothelial cells and blood vessels at the host bone-LS-PEK interface (A) and host bone- βTCP interface (B) showing blood vessels in green and nuclei counterstained in blue. Positive control of sheep lung (C) and negative control of sheep mandible omitting primary antibody (D) are also shown.

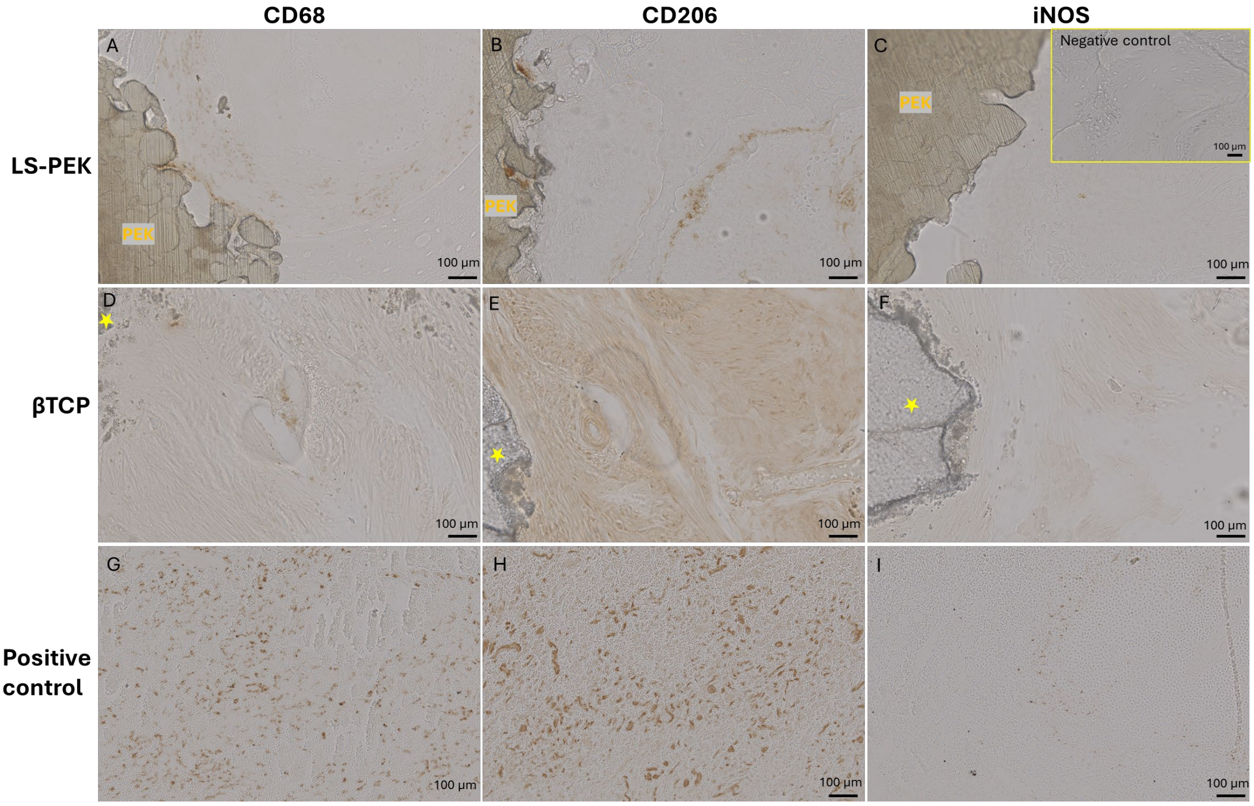

Supplementary Fig. 6: Immunohistochemistry for macrophage markers at tissue interfaces of LS-PEK (A-C) and βTCP constructs (D-F). The level of inflammation was assessed by immunohistochemistry (IHC) using key macrophage markers: CD68 (pan-macrophage marker, ab125212), CD206 (M2-like, anti-inflammatory marker, ab64693), and iNOS (M1-like, pro-inflammatory marker, ab15323)**.** βTCP constructs are labelled by the stars. Positive control of sheep spleen (G-I) and negative control of sheep mandible tissue (C-insert) are also shown. The macrophage response characterized by these markers demonstrated low overall inflammatory response to the implanted scaffolds.

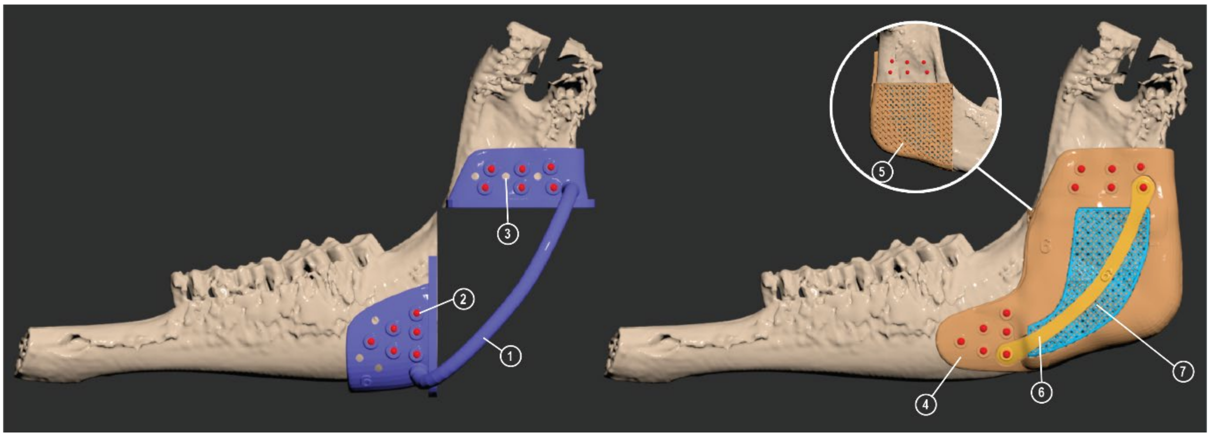

Supplementary Fig. 7: Virtual surgical planned cutting guide and hybrid artificial bone. (1) LS nylon-12 surgical guide design (2) Screw hole locations for LS-PEK frame (3) Screw hole locations for surgical guide (4) LS-PEK frame (5) TPMS gyroid architecture of the middle component of the LS-PEK frame (6) LS-PEK crossbar securing βTCP lattice (7) βTCP lattice inset into LS-PEK frame.

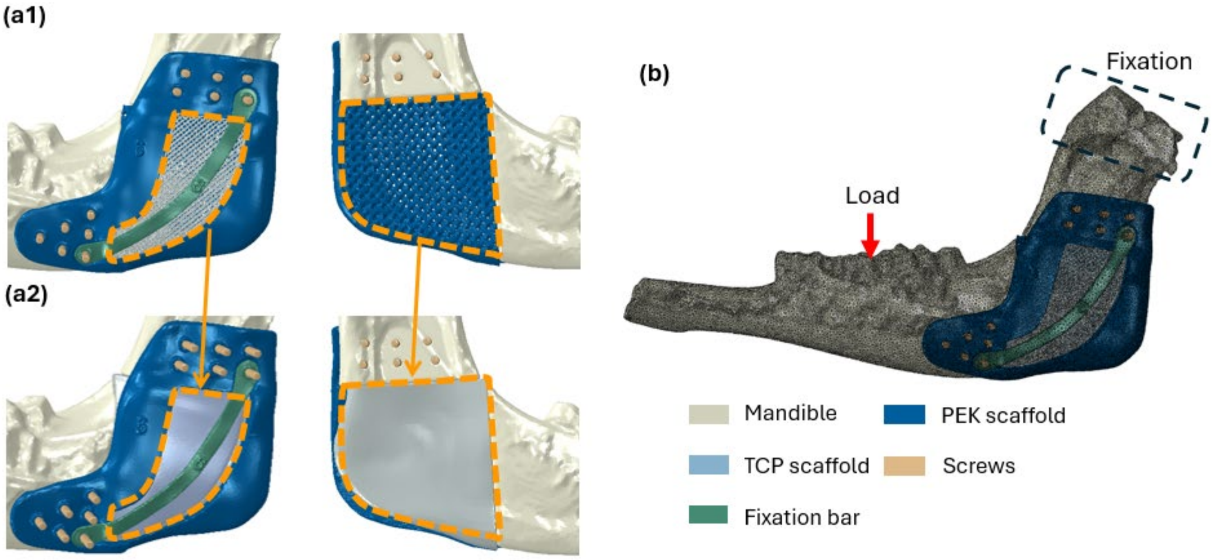

Supplementary Fig. 8: Mechanobiological numerical model of hybrid artificial bone. (**a1**) The βTCP scaffold was positioned on the lateral side of the LS-PEK frame, while the LS-PEK TPMS scaffold component of the frame was defined on the medial side. (**a2**) The homogenized solid model of the LS-PEK and βTCP scaffolds were tailored for the bone growth simulation. (**b**) The mandible was fixed at the condyle, and a compressive load was applied to the teeth for testing.

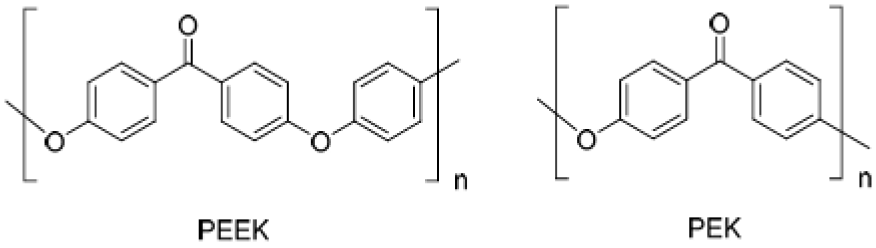

Supplementary Fig. 9: Chemical structure of PEEK and PEK.

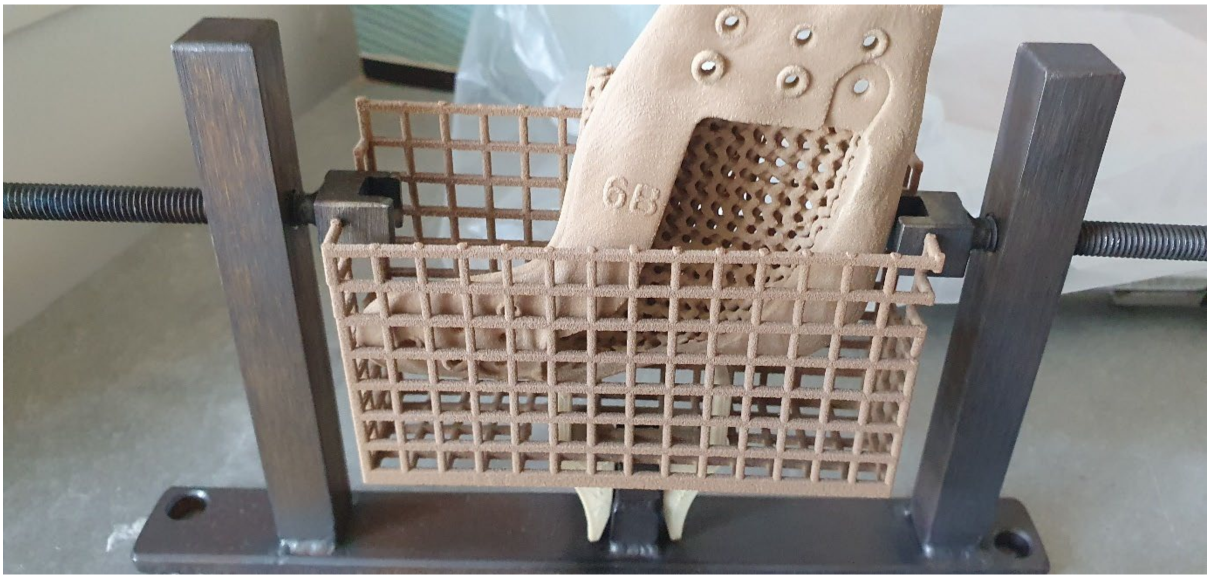

Supplementary Fig. 10: A custom LS-PEK frame held in the thermal toughening tray.

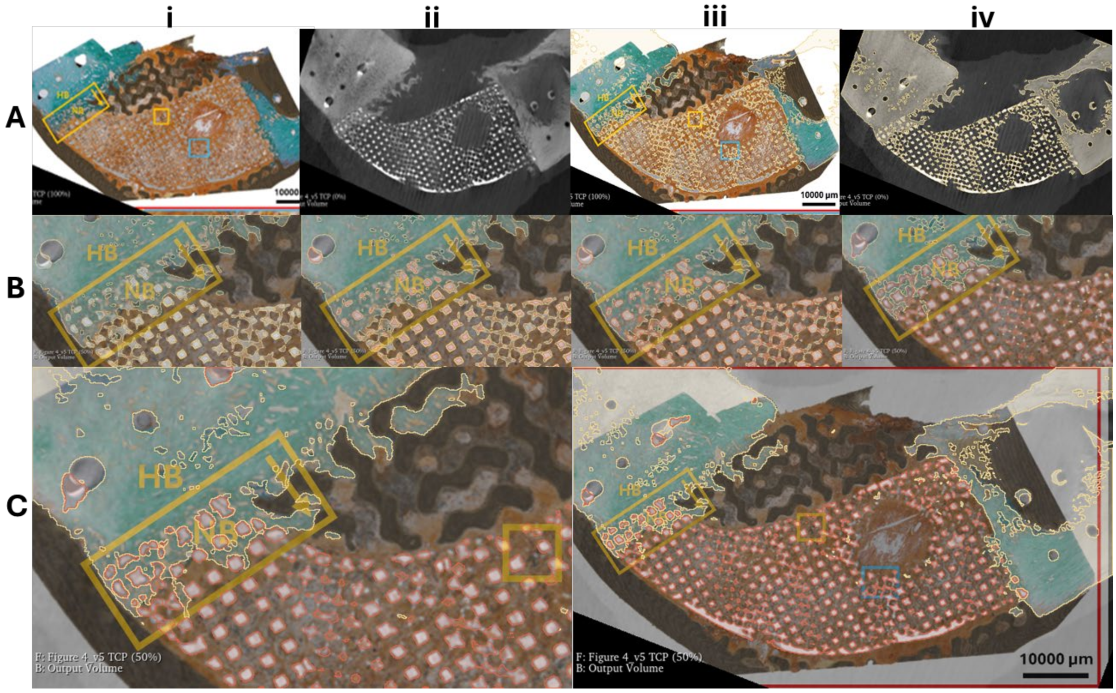

Supplementary Fig. 11a: Segmentation process for quantification of bone in βTCP scaffold implants.

A. Alignment of histology (i) and µCT (ii) to validate segmentation-histology overlap (iii). The final thresholding of µCT (iv).
B. Segmentation of Bone with βTCP from Bone within βTCP lattice by: thresholding bone (i), thresholding βTCP core (ii), applying a geometric offset to βTCP and subtraction of βTCP from Bone (iii) before filtering bone segmentation for thickness and island size (iv).
C. Segmentation Results. Close up (left), Overall (right)

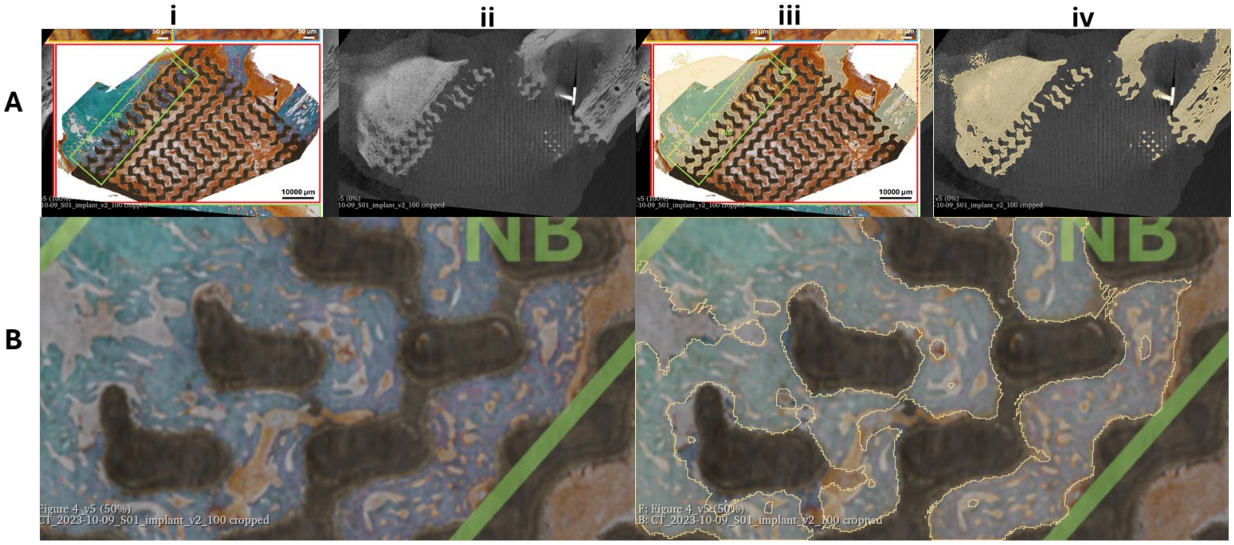

Supplementary Fig. 11b: Segmentation process for quantification of bone in the LS-PEK scaffold implants.

A. Alignment of histology (i) and µCT (ii) to validate segmentation-histology overlap (iii). The final thresholding of µCT (iv).
B. Segmentation Results. High magnification view of histology (left). Outlined segmented bone validated by histology (right).

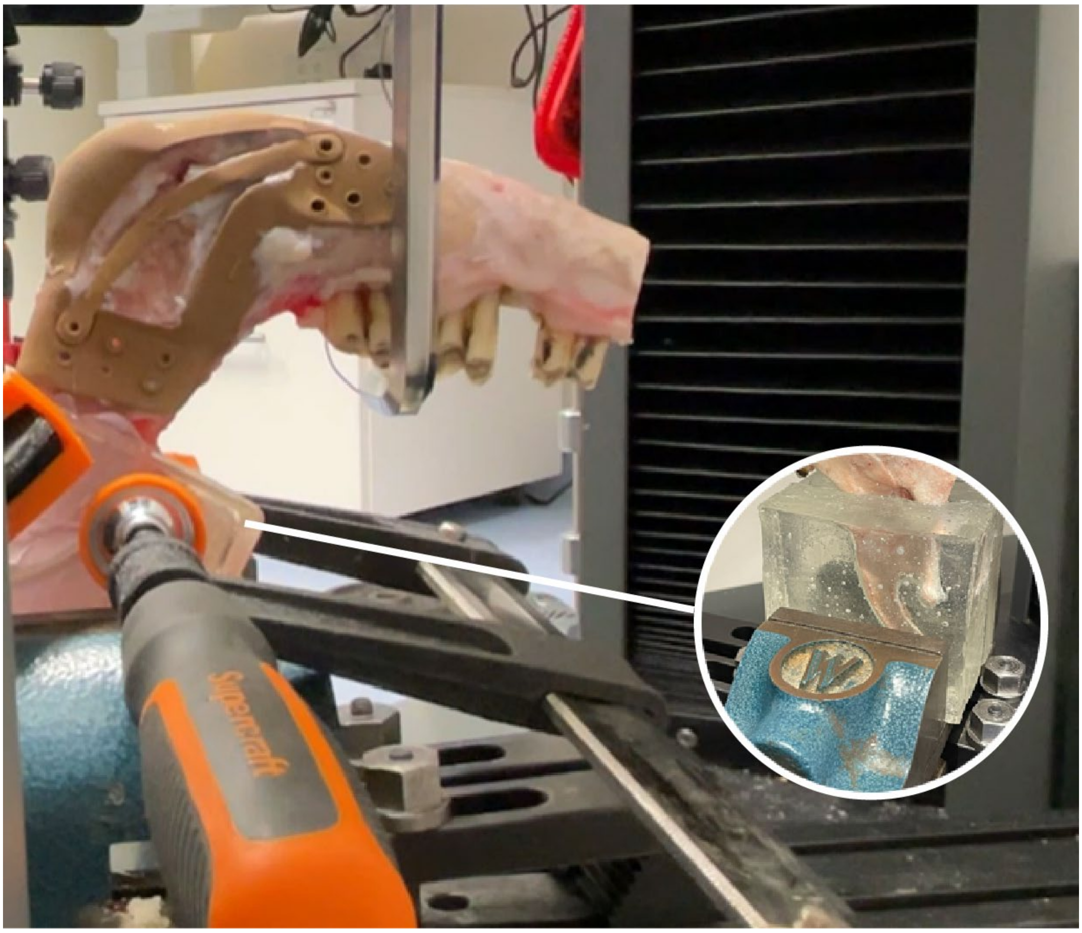

Supplementary Fig. 12: Mechanical testing setup of the harvested hemimandible with hybrid artificial bone after removal of fixation screws and immobilized using custom resin condyle and coronoid moulds. The molar teeth were loaded until the first sign of mandibular system failure, and force-displacement curves were generated.

Supplementary Table 1. The parameters for printing PEK using the EOS P800 3D-printer system

Layer thickness (mm)	0.12
Temperature set points (℃)<br>Process chamber<br>Building platform<br>Exchangeable frame	 364<br>336<br>343
Post-sintering time (s)	12
Beam offset (mm)	0.41
Exposure Parameters Set	Custom
Cool-down cycle	High-Temp Cooldown
Build Volume	350mm x 230mm
Base powder thickness before warmup (mm)	2.5
Base powder thickness after warmup (mm)	6

Supplementary Table 2. The parameters used for the post printing quenching process

Parameter	Value
Heat soak temperature (℃)	300±5
Heat soak time (s)	240
Quench time (s)	50
Quench air temperature (℃)	23±4
Quench air flow rate (m^3/min)	17±10
