# Supplementary material for: Mechanobiologically-optimized non-resorbable artificial bone for patient-matched scaffold-guided bone regeneration
Source: Nat Commun. 2025 Oct 24;16:9422. doi: 10.1038/s41467-025-64466-z (PMC12552697; doi:10.1038/s41467-025-64466-z)
Supplement: Supplementary file 2 — Description of Additional Supplementary Files [file 41467_2025_64466_MOESM2_ESM.pdf]

Title: Supplementary Movie 1
Description: Manufacturing and implantation of the artificial bone implant in sheep.

Title: Supplementary Movie 2
Description: Chewing Behavior of sheep post 6 months of surgical implantation

Title: Supplementary Movie 3
Description: Stress-driven CBCT bone growth patterns
